# Ruthenium Picolinate Complex as a Redox Photosensitizer With Wide-Band Absorption

**DOI:** 10.3389/fchem.2019.00327

**Published:** 2019-05-14

**Authors:** Yusuke Tamaki, Kazuma Tokuda, Yasuomi Yamazaki, Daiki Saito, Yutaro Ueda, Osamu Ishitani

**Affiliations:** Department of Chemistry, Tokyo Institute of Technology, Tokyo, Japan

**Keywords:** redox photosensitizer, CO_2_ reduction, photocatalyst, Ruthenium(II) complex, wide-band absorption

## Abstract

Ruthenium(II) picolinate complex, [Ru(dmb)_2_(pic)]^+^ (**Ru(pic)**; dmb = 4,4′-dimethyl-2,2′-bipyridine; Hpic = picolinic acid) was newly synthesized as a potential redox photosensitizer with a wider wavelength range of visible-light absorption compared with [Ru(N^∧^N)_3_]^2+^ (N^∧^N = diimine ligand), which is the most widely used redox photosensitizer. Based on our investigation of its photophysical and electrochemical properties, **Ru(pic)** was found to display certain advantageous characteristics of wide-band absorption of visible light (λ_abs_ < 670 nm) and stronger reduction ability in a one-electron reduced state ($E_{1/2}^{\text{red}}$ = −1.86 V vs. Ag/AgNO_3_), which should function favorably in photon-absorption and electron transfer to the catalyst, respectively. Performing photocatalysis using **Ru(pic)** as a redox photosensitizer combined with a Re(I) catalyst reduced CO_2_ to CO under red-light irradiation (λ_ex_ > 600 nm). TON_CO_ reached 235 and Φ_CO_ was 8.0%. Under these conditions, [Ru(dmb)_3_]^2+^ (**Ru(dmb)**) is not capable of working as a redox photosensitizer because it does not absorb light at λ > 560 nm. Even in irradiation conditions where both **Ru(pic)** and **Ru(dmb)** absorb light (λ_ex_ > 500 nm), using **Ru(pic)** demonstrated faster CO formation (TOF_CO_ = 6.7 min^−1^) and larger TON_CO_ (2347) than **Ru(dmb)** (TOF_CO_ = 3.6 min^−1^; TON_CO_ = 2100). These results indicate that **Ru(pic)** is a superior redox photosensitizer over a wider wavelength range of visible-light absorption.

## Introduction

Redox photosensitizers, which absorb visible light and facilitate the electron transfer process, play a key role in various photochemical reactions, such as CO_2_ reduction (Takeda et al., 2017; Tamaki and Ishitani, 2017), water oxidation (Fukuzumi et al., 2016), hydrogen evolution (Schulz et al., 2012), and organic synthesis (Prier et al., 2013). Effective photosensitizers should be endowed with three important properties, including (1) visible-light absorption, (2) a long lifetime in the excited state to initiate the electron transfer process, and (3) reducing and/or oxidizing power that is strong enough to donate electrons or holes to the catalyst. In particular, the utilization of visible-light over a wider range of wavelengths is important both to utilize sunlight efficiently and avoid the internal filter effect and side reactions that are commonly caused by the light-absorption of catalysts and/or electron donor/acceptor. Ru(II) complexes coordinated with three diimine ligands, [Ru(N^∧^N)_3_]^2+^ (N^∧^N = diimine ligand) are the most widely used redox photosensitizers in various photochemical redox reactions because these types of complexes exhibit strong absorption in the visible-light region and have a long lifetime in their triplet metal-to-ligand charge-transfer (^3^MLCT) excited states (Juris et al., 1988; Thompson et al., 2013).

However, one of the disadvantages of [Ru(N^∧^N)_3_]^2+^-type photosensitizers is the limited access to the wavelength region of visible light, e.g., λ_abs_ < 560 nm in the cases of N^∧^N = 2,2′-bipyridine (bpy) and 4,4′-dimethyl-2,2′-bipyridine (dmb), and these complexes cannot utilize visible light having lower energy (λ > 560 nm). To overcome this, ligand-modified Ru(II) photosensitizers have been reported. For example, Ru(II) complexes have an extended π-system for photodynamic therapy (Zhang et al., 2017) and multinuclear Ru(II) complexes by conjugated bridging ligand are used for hydrogen evolution (Tsuji et al., 2018). However, these modifications lower the reducing power of photosensitizers and limit the choice of catalyst especially for the reduction of CO_2_. On the other hand, we have reported an osmium(II) analog, i.e., [Os(N^∧^N)_3_]^2+^, which could function as a redox photosensitizer utilizing a much wider wavelength range of visible light (λ_abs_ < 700 nm) due to its singlet-to-triplet direct excitation (S-T absorption) and drive photocatalytic CO_2_ reduction by red-light irradiation (λ_ex_ > 620 nm) in the combination with rhenium(I) catalyst unit (Tamaki et al., 2013b), whereas the high toxicity of Os^VIII^O_4_ inhibits the wider application of osmium complexes.

**Chart 1:**
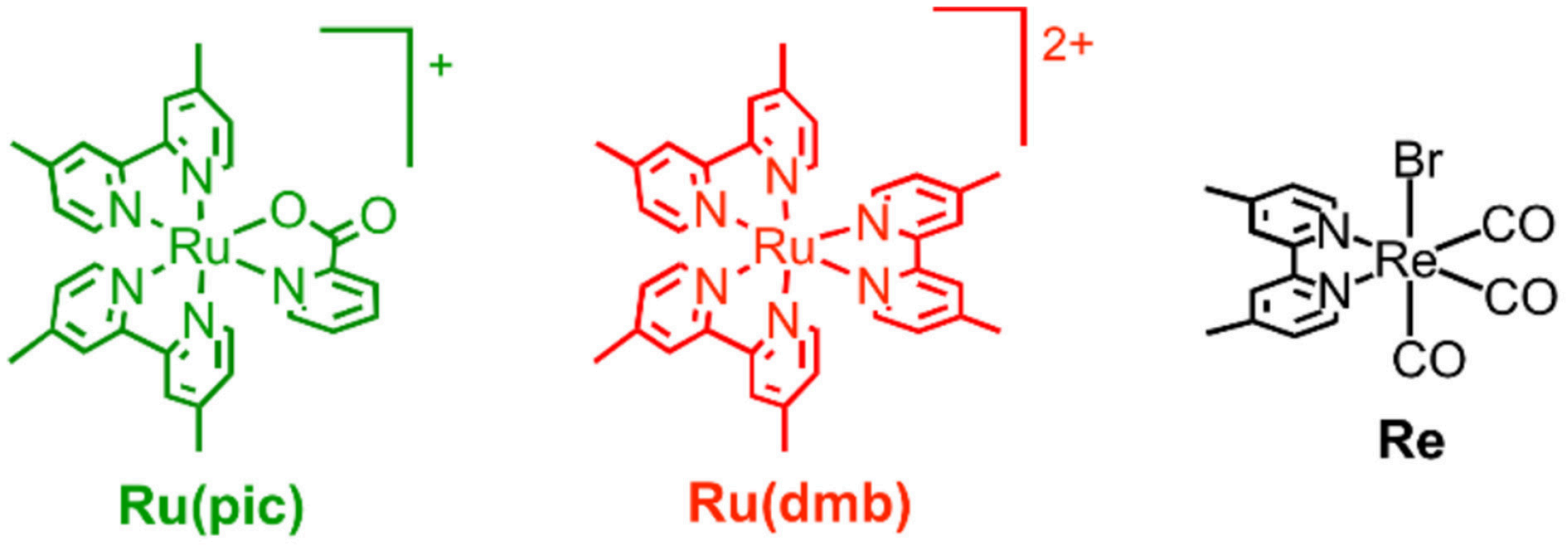
Structures and abbreviations of complexes used.

Therefore, we developed a novel ruthenium(II) redox photosensitizer that can utilize a wider wavelength range of visible light than [Ru(N^∧^N)_3_]^2+^. In the photocatalytic system for CO_2_ reduction, a photosensitizer mediates an electron from a sacrificial electron donor to a catalyst. Since the positive shift of the LUMO level of redox photosensitizer should limit the choice of a catalyst for reducing CO_2_, for the expansion of the useable wavelength range, we try to decrease the energy-gap between HOMO and LUMO by the negative shift of the HOMO level, while maintaining the LUMO level. We introduced anionic electron-donating picolinate instead of a diimine ligand into a ruthenium complex (Norrby et al., 1997; Couchman et al., 1998). [Ru(dmb)_2_(pic)]^+^ (**Ru(pic)**; Hpic = picolinic acid) was synthesized, and we investigated its photophysical properties and functions as a redox photosensitizer using [Ru(dmb)_3_]^2+^ (**Ru(dmb)**) as a reference redox photosensitizer and Re(dmb)(CO)_3_Br (**Re**) as a catalyst for the reduction of CO_2_ (Hawecker et al., 1983; Gholamkhass et al., 2005; Tamaki et al., 2016). Chart 1 shows structures and abbreviations of the metal complexes used.

## Results and Discussion

**Figure 1:**
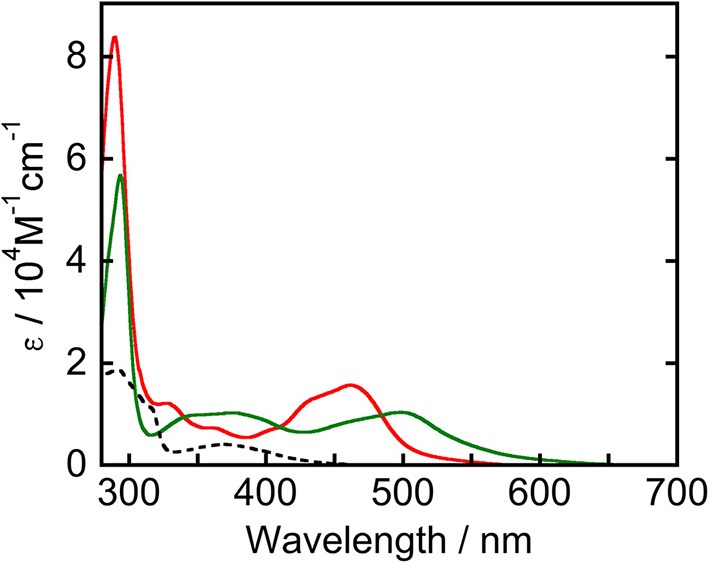
UV-vis absorption spectra of **Ru(pic)** (green line), **Ru(dmb)** (red line), and **Re** (broken line) measured in a DMA solution.

Figure 1 displays UV-vis absorption spectra of **Ru(pic)**, **Ru(dmb)**, and **Re** measured in *N*,*N*-dimethylacetamide (DMA). **Ru(pic)** exhibited a broad singlet MLCT absorption band at λ_abs_ = 450–640 nm, with molar absorptivity at an absorption maximum (λ_max_ = 498 nm) of 1.04 × 10^4^ M^−1^cm^−1^, which was red-shifted in wavelength compared to that of **Ru(dmb)** (λ_abs_ = 420–550 nm). The absorption band attributed to the π-π^*^ transition of dmb ligands was observed at 294 nm. According to this result, **Ru(pic)** have the potential to utilize visible light over a wider range of wavelengths (λ_abs_ < 670 nm) than **Ru(dmb)** (λ_abs_ < 560 nm). This expected red-shift of the MLCT band should be induced by the stronger electron-donating ability of the picolinate ligand to negatively shift the energy level of HOMO.

**Figure 2:**
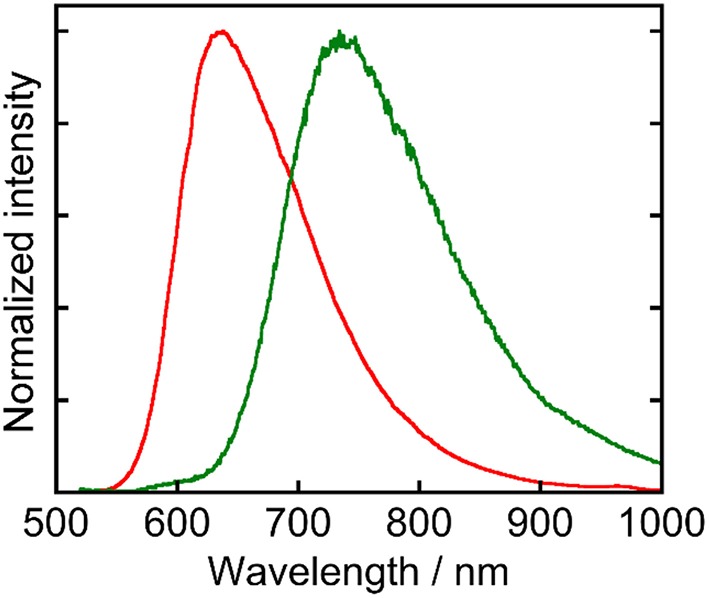
Normalized emission spectra of **Ru(pic)** (green line) and **Ru(dmb)** (red line) measured in a DMA solution. The excitation wavelength was 480 nm.

**Ru(pic)** exhibited phosphorescence from its ^3^MLCT excited state (Figure 2) with a quantum yield of Φ_em_ = 0.8% and a lifetime of τ_em_ = 66 ns. Emission spectrum of **Ru(pic)** (λ_em_ = 734 nm) was also red-shifted compared to that of **Ru(dmb)** (λ_em_ = 638 nm). The quantum yield and lifetime of **Ru(pic)** were smaller and shorter than those of **Ru(dmb)** (Φ_em_ = 9.1%, τ_em_ = 741 ns) due to the 12-times faster non-radiative deactivation process (**Ru(pic)**: *k*_nr_ = 1.5 × 10^7^ s^−1^; **Ru(dmb)**: *k*_nr_ = 1.2 × 10^6^ s^−1^), which is a reasonable behavior from energy-gap law. Table 1 summarizes photophysical properties of **Ru(pic)** along with those of **Ru(dmb)** and **Re**.

**Table 1:** Photophysical properties of **Ru(pic)**, **Ru(dmb)**, and **Re**.*^a^*.

**Complex**	**λ_abs_/nm (*ε*/10^4^M^−1^cm^−1^)**		**λ_em_*^b^*/nm**	**Φ_em_*^b^***	**τ_em_*^c^*/ns**	***k*_r_*^d^*/10^5^s^−1^**	***k*_nr_*^e^*/10^6^s^−1^**	***E*_00_*^f^*/eV**
	π-π^*^	**^**1**^MLCT**						
**Ru(pic)**	294 (5.67)	498 (1.04)	734	0.008	66	1.2	15	1.75
**Ru(dmb)**	290 (8.38)	462 (1.57)	638	0.091	741	1.2	1.2	2.02
**Re**	292 (1.87)	370 (0.41)	–	–	–	–	–	–

a Measured in DMA.

b Excitation wavelength: 480 nm.

c Excitation wavelength: 510 nm.

d Rate constants for radiative deactivation calculated as k_r_ = Φ_em_/τ_em_.

e Rate constants for non-radiative deactivation calculated as k_nr_ = (1–Φ_em_)/τ_em_.

f *Energy for 0-0 transition obtained from Franck-Condon analyses of the emission spectra*.

**Figure 3:**
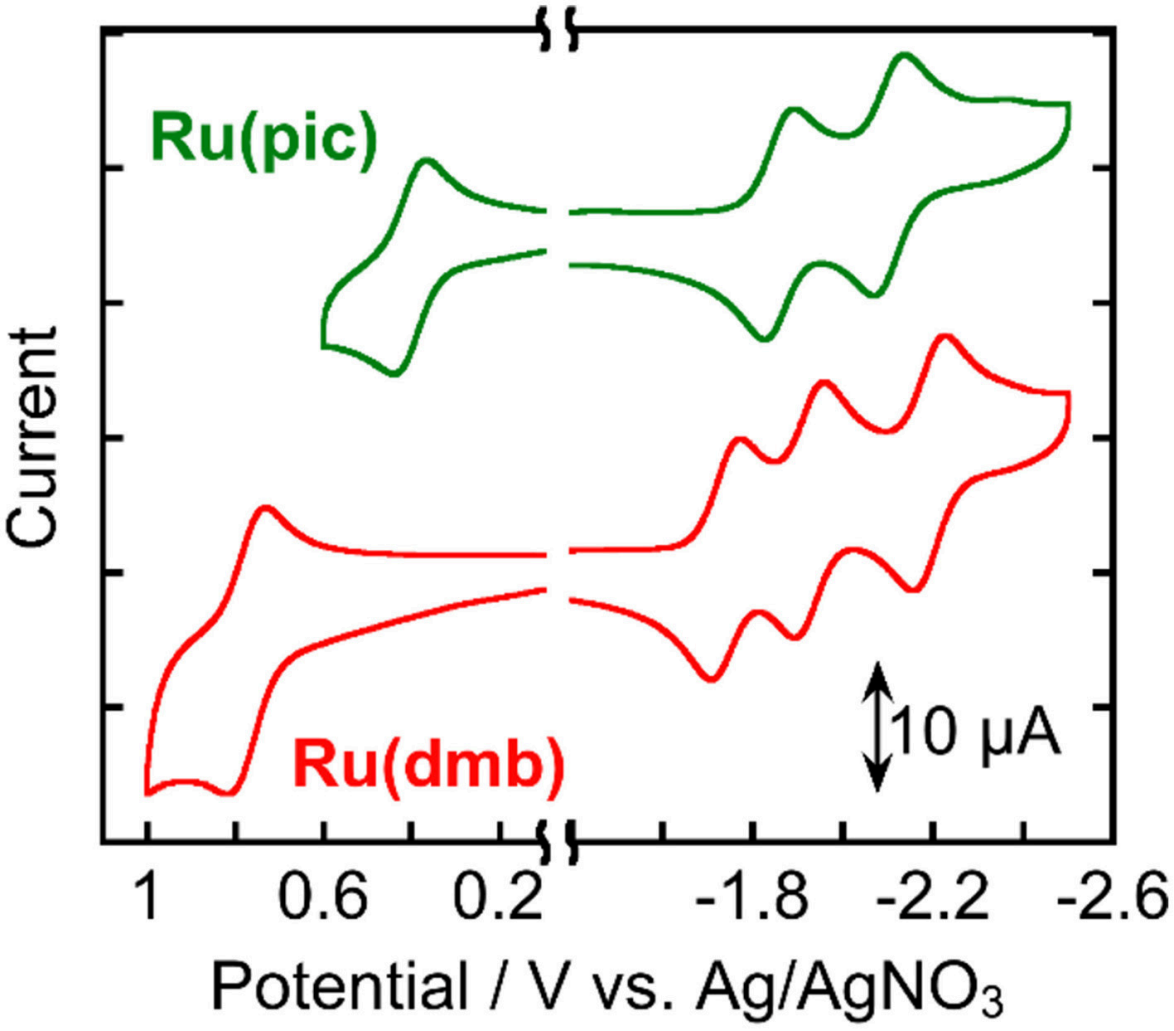
Cyclic voltammograms of **Ru(pic)** and **Ru(dmb)** measured in a DMA solution containing Et_4_NBF_4_ (0.1 M) as a supporting electrolyte with a Ag/AgNO_3_ (10 mM) reference electrode.

Figure 3 shows the cyclic voltammograms of **Ru(pic)** and **Ru(dmb)** and their redox potentials are summarized in Table 2 along with that of **Re**. **Ru(pic)** displayed two reversible reduction waves and a reversible oxidation wave, which are attributable to the subsequent reduction of two dmb ligands and the oxidation couple of Ru^III/II^, respectively. Both the first reduction ($E_{1/2}^{\text{red}}$ = −1.86 V vs. Ag/AgNO_3_) and oxidation ($E_{1/2}^{0\text{x}}$ = 0.41 V) waves were observed at more negative potentials than those of **Ru(dmb)** ($E_{1/2}^{\text{red}}$ = −1.74 V and $E_{1/2}^{0\text{x}}$ = 0.77 V), which should be induced by the stronger electron-donating ability of the picolinate ligand. The stronger reducing power of one-electron reduced species (OERS) of **Ru(pic)** ($E_{1/2}^{\text{red}}$ = −1.86 V) facilitates an increase in the number of choices of applicable catalyst because the electron transfer from OERS of **Ru(pic)** to a catalyst must occur during photocatalysis in the case of reductive quenching mechanisms. When using **Ru(pic)** as a photosensitizer and **Re** as a catalyst, the electron transfer process from OERS of **Ru(pic)** to **Re** ($E_{1/2}^{\text{red}}$ = −1.76 V) occurs exothermically.

**Table 2:** Electrochemical properties of the metal complexes in DMA*^a^*.

**Complex**	***E*_1/2_/V vs. Ag/AgNO_3_ (Δ*E/*mV)**				*E*(PS^+^/PS*)*^b^*/ V	*E*(PS*/PS^−^)*^b^*/ V
	**Ru^**III/II**^**	**M(N^∧^N/N^∧^N·^−^) (M = Ru or Re)**				
**Ru(pic)**	+0.41 (72)	−1.86 (72)	−2.11 (69)	–	−1.34	−0.11
**Ru(dmb)**	+0.77 (68)	−1.74 (72)	−1.93 (70)	−2.19 (74)	−1.25	+0.28
**Re**	–	−1.76 (74)	–	–	–	–

a Measured in a DMA solution containing the complex (0.5 mM) and Et_4_NBF_4_ (0.1 M) with a scan rate of 200 mV·s^−1^ under an Ar atmosphere.

b *Redox potentials of the photosensitizers (PS) in their excited states were calculated from E${}_{1/2}^{0x}$-E_00_ and E${}_{1/2}^{red}$ + E_00_, respectively*.

**Figure 4:**
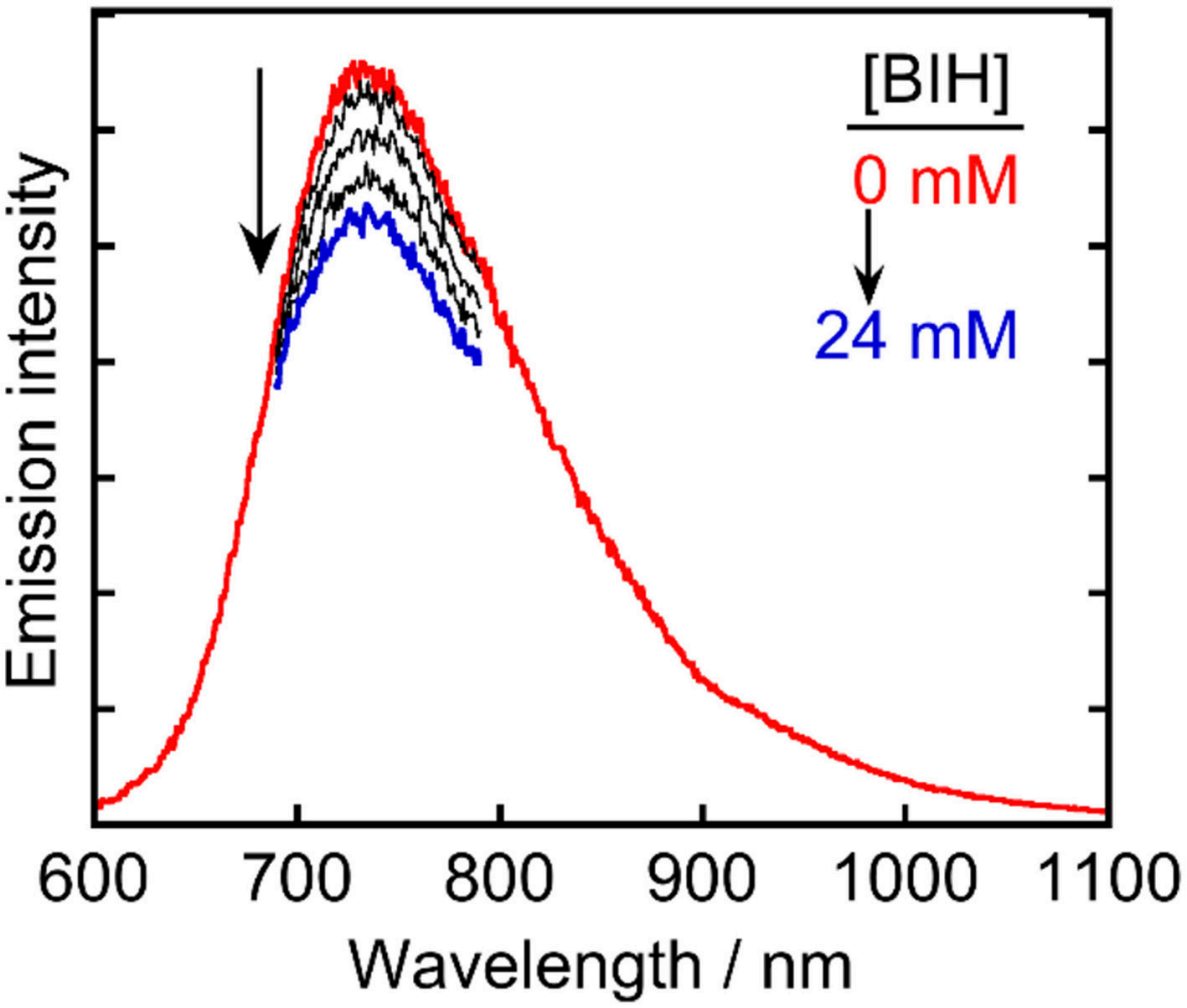
Emission spectra of **Ru(pic)** in Ar-saturated DMA-TEOA (5:1 v/v) containing five different concentrations of BIH (0–24 mm).

These results indicated that **Ru(pic)** had some advantages with respect to its function as a redox photosensitizer compared with **Ru(dmb)**, including its wider wavelength range of visible-light absorption and stronger reducing power of OERS, which is effective in the electron transfer to the catalyst. However, certain unfavorable properties were also observed, i.e., a shorter lifetime (τ_em_ = 66 ns) and weaker oxidizing power in its excited state (Δ*E* = *E*(Ru(dmb)^*^/**Ru(dmb)^−^**)–*E*(Ru(pic)^*^/**Ru(pic)^−^**) = 0.28–(−0.11) = 0.39 V). In the reductive quenching process, an excited photosensitizer accepts an electron from a sacrificial electron donor. Weaker oxidation power in the excited state of a photosensitizer should decrease the driving force of this electron transfer process. In addition, since this process competes with the radiative and non-radiative deactivation processes from the excited state of a photosensitizer by itself, the shorter lifetime results in less opportunity of the reductive quenching process to occur. To evaluate whether reductive quenching occurs, the emission intensity from **Ru(pic)** was compared in the presence of five different concentrations of a sacrificial electron donor, 1,3-dimethyl-2-phenyl-2,3-dihydro-1*H*-benzo[*d*]imidazole (BIH) (Tamaki et al., 2013a; Hasegawa et al., 2015) in DMA-triethanoamine (TEOA; 5:1 v/v). As shown in Figure 4, the emission intensities from the ^3^MLCT excited state of **Ru(pic)** decreased at higher concentrations of BIH, which indicated that the excited **Ru(pic)** was quenched by BIH. The quenching rate constant was determined to be *k*_q_ = 1.7 × 10^8^ M^−1^s^−1^ from the Stern-Volmer plot (Figure S1) and the lifetime of the emission (τ_em_ = 66 ns), which was 8-times slower than that of **Ru(dmb)** (*k*_q_ = 1.4 × 10^9^ M^−1^s^−1^) as expected from the weaker oxidizing power in the ^3^MLCT excited state of **Ru(pic)**. In the photocatalytic reaction condition, i.e., [BIH] = 0.2 M, 69% of the excited **Ru(pic)** was estimated to be quenched by BIH, which should be enough to initiate a photocatalytic reaction.

**Figure 5:**
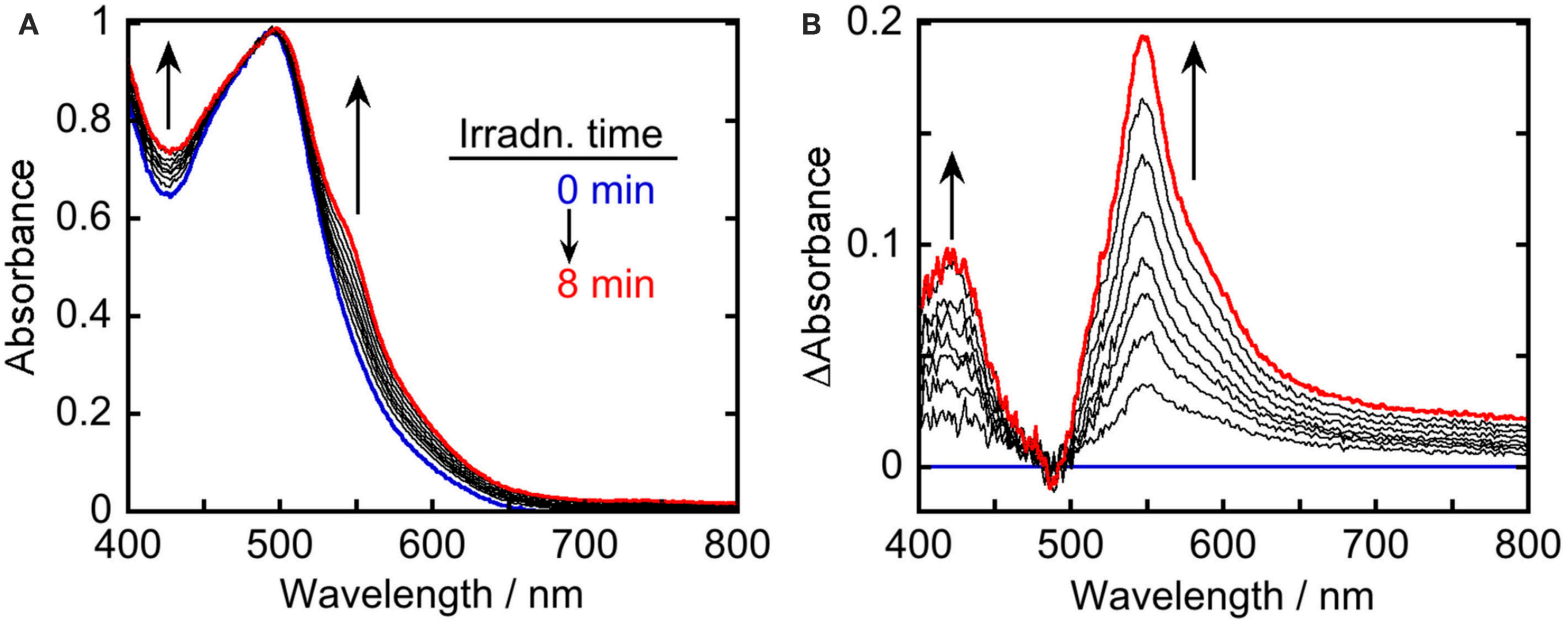
UV-vis **(A)** absorption and **(B)** differential absorption spectral change of a DMA-TEOA (5:1 v/v, 4 mL) solution containing **Ru(pic)** (0.1 mM) and BIH (0.2 M) during irradiation using light at λ_ex_ = 480 nm (0–8 min at 1-min intervals). The incident light intensity was 5.0 × 10^−9^ einstein·s^−1^. Blue and red lines represent spectra at 0 and 8-min irradiation, respectively.

To clarify the produced species as a result of the quenching of excited **Ru(pic)** by BIH, UV-vis absorption spectral change was observed during photo-irradiation of **Ru(pic)** in the presence of BIH (Figure 5). Irradiation by light at λ_ex_ = 480 nm caused spectral changes and new absorption bands appeared at λ_abs_ = 420 and 547 nm. The shape of differential absorption spectra before and after irradiation (Figure 5B) were quite similar to that of OERS of **Ru(pic)** obtained by electrochemical spectroscopy (Figure S2). These results indicate that the reductive quenching of the ^3^MLCT excited state of **Ru(pic)** by BIH proceeded successfully to give OERS of **Ru(pic)** (Equation 1) and **Ru(pic)** can be expected to function as a redox photosensitizer over the wide-range absorption of visible light.

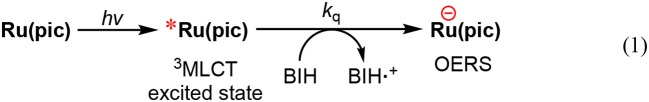



**Figure 6:**
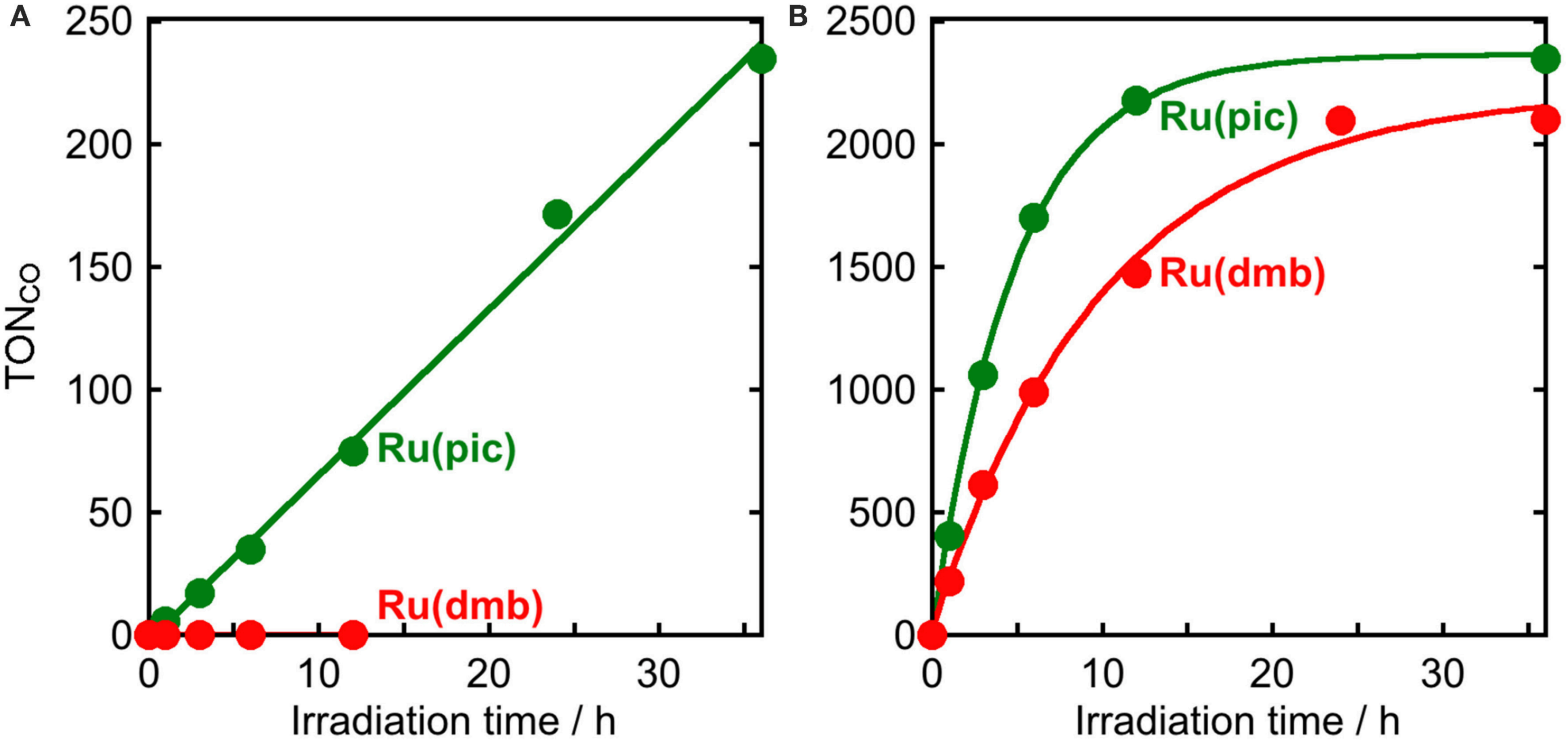
Photocatalytic production of CO as a function of irradiation time using **Ru(pic)** (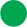) or **Ru(dmb)** (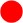) as a photosensitizer: CO_2_-saturated DMA-TEOA (5:1 v/v, 2 mL) solutions containing Ru(II) photosensitizer (50 μM), **Re** (50 μM), and BIH (0.2 M) were irradiated at **(A)** λ_ex_ > 620 nm or **(B)** λ_ex_ > 500 nm.

The results of photocatalytic reactions for the reduction of CO_2_ are summarized in Table 3. In a typical run of photocatalytic reactions, a mixed solution of DMA-TEOA (5:1 v/v) containing **Ru(pic)** (50 μM), **Re** (50 μM), and BIH (0.2 M) as a sacrificial electron donor was irradiated under a CO_2_ atmosphere using light at λ_ex_ > 620 nm. CO production proceeded linearly and selectively and the turnover number for CO production (TON_CO_) was 235 after 36 h of irradiation (Figure 6A). The quantum yield for CO formation (Φ_CO_) was determined to be Φ_CO_ = 8% using λ_ex_ = 600-nm light (light intensity: 6.0 × 10^−9^ einstein·s^−1^). By contrast, when using **Ru(dmb)** as a redox photosensitizer instead of **Ru(pic)**, no photocatalysis proceeded (Figure 6A) because **Ru(dmb)** does not absorb lower-energy light at λ_ex_ > 620 nm (Figure 1). To compare the function as a redox photosensitizer, the photocatalytic reactions were also conducted under photo-irradiation condition, where both **Ru(pic)** and **Ru(dmb)** absorb incident light (λ_ex_ > 480 nm). In this condition, both systems photocatalytically produced CO with high selectivity. Figure 6B shows the time course of photocatalytic CO production using light at λ_ex_ > 500 nm, and the system using **Ru(pic)** formed CO faster (TOF_CO_ = 6.7 min^−1^) than **Ru(dmb)** (TOF_CO_ = 3.6 min^−1^) in the initial stage of photocatalysis. TON_CO_ reached 2347 and 2100 after 36 h of irradiation using **Ru(pic)** and **Ru(dmb)**, respectively. The values of Φ_CO_ using light at λ_ex_ = 480 nm (light intensity: 6.0 × 10^−9^ einstein·s^−1^) were 10% and 44% in the cases using **Ru(pic)** and **Ru(dmb)**, respectively. The **Ru(pic)** system demonstrated similar Φ_CO_ values in both irradiation conditions (λ_ex_ = 600 and 480 nm). These results indicated that **Ru(pic)** has a clear advantage of a wider wavelength range of utilizable visible light compared to **Ru(dmb)**, even for the photocatalytic condition of λ_ex_ > 480 nm. Since **Ru(pic)** displays larger molar absorptivity in the λ_abs_ > 480-nm region and a wider wavelength range than **Ru(dmb)** (Figure 1), **Ru(pic)** absorbs a much larger number of photons at λ_ex_ > 480-nm, which leads to a faster TOF_CO_ and larger TON_CO_, even though the quantum yields for CO production were lower.

**Table 3:** Photocatalytic properties using the mixed system of the Ru(II) photosensitizer and **Re***^a^*.

**Photosensitizer**	**Wavelength**	**TON*^b^***			**Φ_CO_*^f^*/%**	***k*_q_*^i^***	**η_q_*^j^*/%**	**Φ_OERS_*^k^*/%**
		**CO**	**HCOOH**	**H_**2**_**		**10^**8**^ M^**−1**^s^**−1**^**		
**Ru(pic)**	λ_ex_ > 600 nm	235*^c^*	4*^c^*	< 1*^c^*	8.0*^g^*	1.7	69	–
**Ru(dmb)**		n.d.*^c^^,^^e^*	n.d.*^c^^,^^e^*	n.d.*^c^^,^^e^*	–	–	–	–
**Ru(pic)**	λ_ex_ > 480 nm	2347*^d^*	< 1*^d^*	< 1*^d^*	10*^h^*	1.7	69	8.3
**Ru(dmb)**		2100*^d^*	11*^d^*	< 1*^d^*	44*^h^*	14	99	66

a A CO_2_-saturated DMA-TEOA (5:1 v/v) mixed solution containing the photosensitizer (50 μM), **Re** (50 μM), and BIH (0.2 M) was irradiated.

b Turnover number for the reaction products after 36 h of irradiation calculated as [product (mol)]/[added **Re** (mol)].

c $\lambda_{ex}$ > 620 nm.

d $\lambda_{ex}$ > 500 nm.

e Irradiation for 12 h.

f Quantum yield of CO production calculated as [CO (mol)]/[absorbed photon (einstein)].

g $\lambda_{ex}$ = 600 nm (light intensity: 6.0 × 10^−9^ einstein·s^−1^).

h $\lambda_{ex}$ = 480 nm (light intensity: 6.0 × 10^−9^ einstein·s^−1^).

i Quenching rate constants for emission from Ru(II) photosensitizers by BIH obtained from the slopes of Stern-Volmer plots and lifetimes of excited states.

j Quenching fractions of emission from Ru(II) photosensitizers by 0.2 M of BIH calculated as 0.2k_q_τ_em_/(1 + 0.2k_q_τ_em_).

k *Quantum yield for one-electron reduction of the photosensitizer using light at λ_ex_ = 480 nm (light intensity: 5.0 × 10^−9^ einstein·s^−1^)*.

The quantitative analyses of BIH and its oxidized compound during photocatalysis were conducted in the system using 0.1 M of BIH to simplify the HPLC analyses. As the only oxidized compound of BIH, two-electron oxidized and deprotonated BIH (BI^+^) was observed (Equation 2).

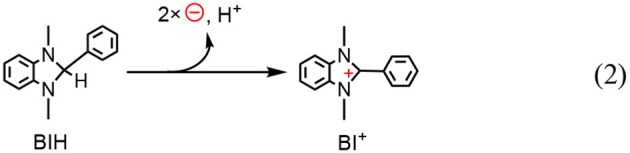



**Figure 7:**
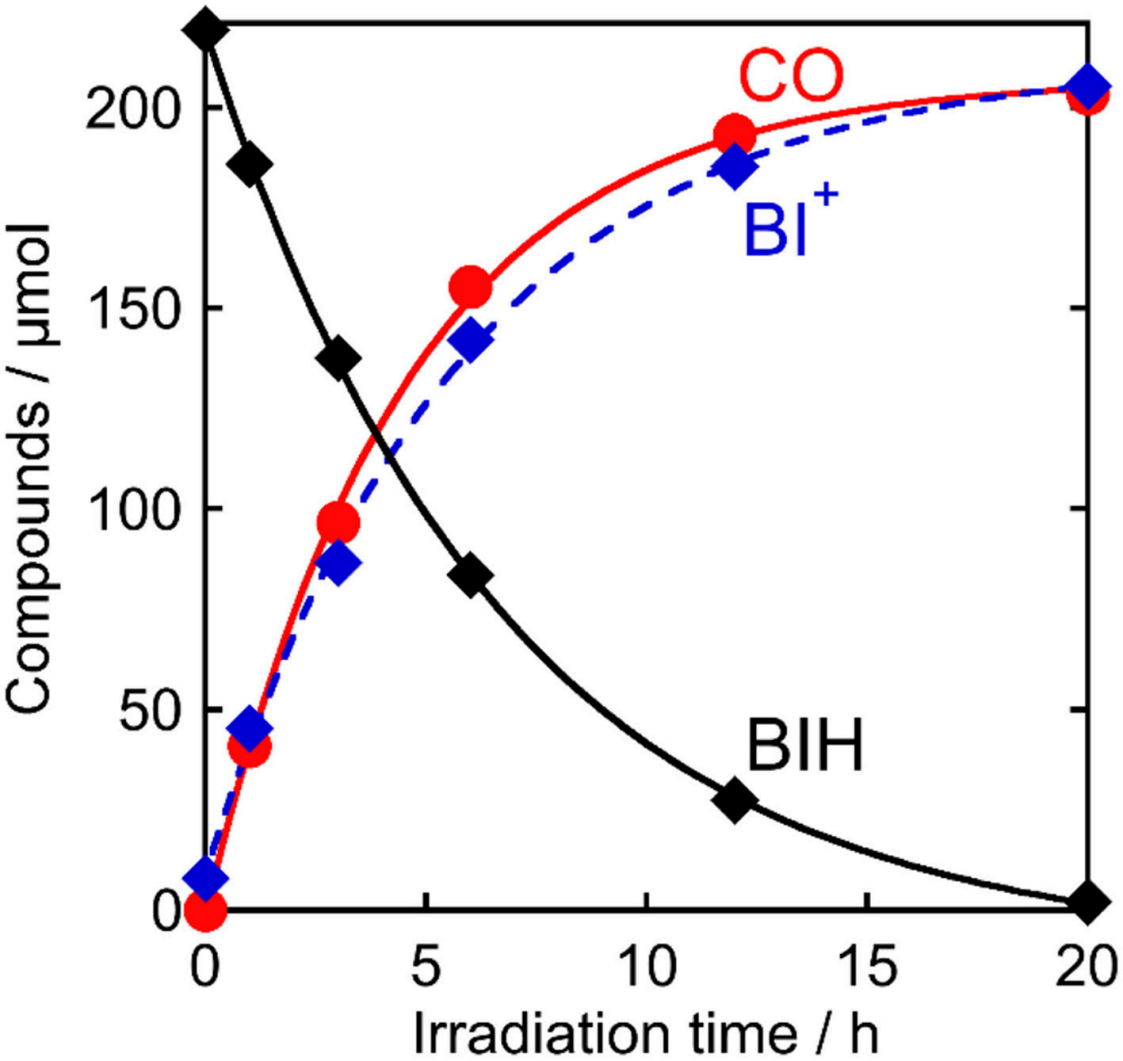
Photocatalytic production of CO (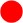) and BI^+^ (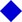) and consumption of BIH (♦): CO_2_-saturated DMA-TEOA (5:1 v/v) solutions containing **Ru(pic)** (50 μM), **Re** (50 μM), and BIH (0.1 M) were irradiated at λ_ex_ > 500 nm.

Figure 7 shows the change in the amounts of both BIH and BI^+^ during photocatalytic reaction along with the amount of CO produced. The amount of produced BI^+^ was fairly similar to that of CO. For example, after 20 h of irradiation, 205 μmol of BI^+^ and 203 μmol of CO formed. CO is the two-electron reduced compound of CO_2_, and BIH supplies two electrons per molecule to give BI^+^ as a oxidized form. These results clearly indicate that BIH acted as a two-electron donor in the photocatalytic reactions using **Ru(pic)** as a redox photosensitizer (Equation 3).

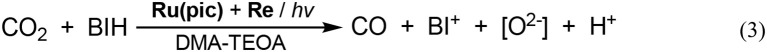



The reaction mechanisms of the photocatalytic reactions using **Ru(pic)** and **Re** were investigated. Since **Re** does not absorb light at λ_ex_ > 460 nm, as shown in Figure 1, **Ru(pic)** should absorb the irradiated photon selectively under photocatalytic reaction conditions, i.e., λ_ex_ > 600 nm or > 480 nm. The photon absorption by **Ru(pic)** gives its OERS via the reductive quenching process of its ^3^MLCT excited state by BIH, as described above (Equation 1). The reducing power of OERS of **Ru(pic)** ($E_{1/2}^{\text{red}}$ = −1.86 V) is strong enough to trigger electron transfer to **Re** ($E_{1/2}^{\text{red}}$ = −1.76 V), which functions as a catalyst for the reduction of CO_2_. The process of two-electron supply using BIH has already been reported in the photocatalytic reaction system using a Ru(II)-Re(I) supramolecular photocatalyst (Tamaki et al., 2013a). The initial process of the photocatalysis is also a photoinduced electron transfer from BIH to the Ru(II) tris-diimine type photosensitizer unit, forming OERS of the photosensitizer unit and one-electron oxidized BIH (BIH**·**^+^). BIH**·**^+^ is rapidly deprotonated by TEOA to give BI**·**. TEOA functioned only as a base, but not as a sacrificial electron donor to quench the excited photosensitizer unit. BI**·** has a strong reducing power ($E_{1/2}^{\text{red}}$ = −1.95 V) (Zhu et al., 2008) enough to provide one more electron to the supramolecular photocatalyst to be converted to BI^+^. In other words, BIH works as a two-electron donor by one-photon excitation of the photocatalyst via the ECE mechanism. Similar processes should also proceed in the photocatalytic system using **Ru(pic)** and **Re** because both **Ru(pic)** ($E_{1/2}^{\text{red}}$ = −1.86 V) and **Re** ($E_{1/2}^{\text{red}}$ = −1.76 V) have a lower reduction potential than BI**·** ($E_{1/2}^{\text{red}}$ = −1.95 V). Based on this investigation, the electron-supply processes of BIH are presumed, as depicted in Equation 4.

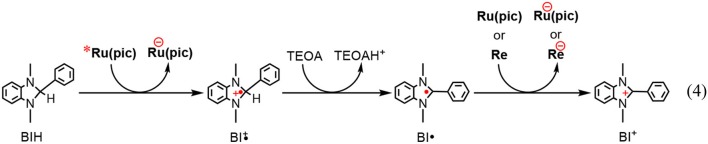



**Scheme 1:**
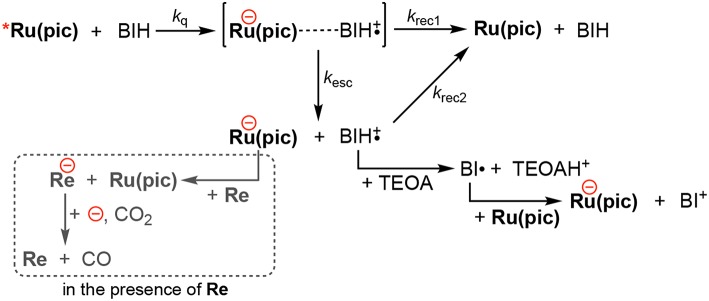
The one-electron reduction processes of **Ru(pic)**.

Photocatalysis using **Ru(pic)** displayed an advantages of a wider wavelength region of visible-light absorption, which achieved both red-light driven CO_2_ reduction (λ_ex_ > 620 nm) and faster CO production than the system using **Ru(dmb)** (λ_ex_ > 500 nm), whereas the quantum yield for CO formation using **Ru(pic)** (Φ_CO_ = 10%) was 1/4 the value when **Ru(dmb)** (Φ_CO_ = 44%) was used. The main reason for smaller Φ_CO_ should be the smaller quantum yield of one-electron reduction (Φ_OERS_) of **Ru(pic)**. Φ_OERS_ of **Ru(pic)** using light at λ_ex_ = 480 nm (light intensity: 5.0 × 10^−9^ einstein·s^−1^) was determined to be 8.3%, which was 1/8 that of **Ru(dmb)** (Φ_OERS_ = 66%). The elementary processes of one-electron reduction of **Ru(pic)** is displayed in Scheme 1. The reductive quenching of the ^3^MLCT excited state of **Ru(pic)** by BIH gives an ion pair, [**Ru(pic)**^−^···BIH**·**^+^]. If the ion pair dissociate, free OERS and BIH**·**^+^ are obtained. The charge-recombination processes from the ion pair or by the re-collision of OERS of **Ru(pic)** and BIH**·**^+^ should form **Ru(pic)** and BIH. The differences in properties between **Ru(pic)** and **Ru(dmb)**, i.e., the cationic valence and the reducing power of OERS, should affect each elementary process and consequently the quantum yield for one-electron reduction. Since OERS of **Ru(dmb)** is a monovalent cation, the ion pair with BIH**·**^+^ involves cationic repulsion, which should accelerate the dissociation process. On the other hand, OERS of **Ru(pic)** is zero-valent, which provides no repulsion between BIH**·**^+^, and therefore, the dissociation process should become slower when using **Ru(pic)** (smaller *k*_esc_). In addition, since the reducing power of OERS of **Ru(pic)** ($E_{1/2}^{\text{red}}$ = −1.86 V) is stronger than that of **Ru(dmb)** ($E_{1/2}^{\text{red}}$ = −1.74 V), the driving forces for the charge-recombination processes become larger when **Ru(pic)** is used (larger *k*_rec1_, *k*_rec2_). Consequently, the smaller Φ_OERS_ using **Ru(pic)** should be induced by the slower dissociation process of the ion pair and the faster charge-recombination processes. The quantitative analyses of the factors controlling Φ_OERS_ of photosensitizing complexes are in progress and will be reported elsewhere.

In the photocatalytic reaction conditions, the electron-consuming process for CO_2_ reduction via the electron transfer to **Re** (the broken box in Scheme 1) will compete against the charge-recombination by the re-collision of OERS and BIH**·**^+^. Therefore, since Φ_OERS_s were determined in the absence of **Re**, Φ_CO_ (10%) was larger than the expected value from half of Φ_OERS_ (8.3/2 = 4.2%), which was derived from the fact that the reduction of CO_2_ to CO is a two-electron reduction process. Higher reduction potential of **Ru(pic)** should operate in favor of the electron transfer to **Re**. Therefore, the ratio of quantum yields for CO_2_ reduction between using **Ru(pic)** and **Ru(dmb)**, i.e., Φ_CO_(**Ru(pic)**)/Φ_CO_(**Ru(dmb)**) = 10/44 = 0.23, became larger than that for one-electron reduction (Φ_OERS_(**Ru(pic)**)/Φ_OERS_(**Ru(dmb)**) = 8.3/66 = 0.13). In other words, **Ru(pic)** has another advantage of faster electron transfer to **Re** in the photocatalysis.

## Experiments

### General Procedures

^1^H NMR spectra were measured using a JEOL ECA400II (400 MHz) system in solutions of acetone-*d*_6_. The residual protons of acetone-*d*_6_ were used as an internal standard for measurements. Electrospray ionization-mass spectroscopy (ESI-MS) was performed using a Shimadzu LCMS-2010A system with acetonitrile as the mobile phase. UV-vis absorption spectra were measured with a JASCO V-565 spectrophotometer. Emission spectra were measured using a Horiba Fluorolog-3-21 spectrofluorometer equipped with a NIR-PMT R5509-43 near infrared detector. A Horiba FluoroCube time-correlated single-photon counting system was used to obtain emission lifetimes. The excitation light source was a NanoLED-515L pulse lamp (510 nm). A HAMAMATSU absolute PL quantum yield spectrometer C9920-02 was used to determine emission quantum yields. The samples were degassed by Ar-bubbling of solutions for 30 min prior to measuring emissions. Emission quenching experiments were performed on solutions containing the complexes and five different concentrations of BIH. The quenching rate constants *k*_q_ were calculated from linear Stern-Volmer plots for the emission from the ^3^MLCT excited state of the photosensitizing complexes and their lifetimes. The redox potentials of the complexes were measured in an Ar-saturated DMA solution containing Et_4_NBF_4_ (0.1 M) as a supporting electrolyte using cyclic voltammetric techniques performed with an ALS CHI-720Dx electrochemical analyzer with a glassy carbon disk working electrode (3 mm diameter), a Ag/AgNO_3_ (10 mM) reference electrode, and a Pt counter electrode. The supporting electrolyte was dried under vacuum at 100°C for 1 day prior to use. The scan rate was 200 mV·s^−1^.

### Photocatalytic Reactions

Photocatalytic reactions were performed in DMA–TEOA (5:1 v/v) solutions containing the photosensitizer (50 μM), **Re** (50 μM), and BIH (0.2 M). After the solution was purged with CO_2_ for 20 min, the solution was irradiated. For TON measurements, the mixed solution (2 mL) in an 11 mL test tube (i.d. 8 mm) was irradiated in a merry-go-round apparatus using λ_ex_ > 620 nm light from a halogen lamp equipped with a Rhodamin B (0.2% w/v, *d* = 1 cm) solution filter or λ_ex_ > 500 nm light from a high-pressure Hg lamp equipped with a uranyl glass and a K_2_CrO_4_ (30% w/w, *d* = 1 cm) solution filter. During irradiation, the temperature of the solution was maintained at 25°C using an EYELA CTP-1000 constant-temperature system. For quantum yield measurements, the mixed solution in a quartz cubic cell (11 mL, light pass length: 1 cm) was irradiated in a Shimadzu photoreaction quantum yield evaluation system QYM-01 using 600 nm or 480 nm light from a 300 W Xe lamp equipped with a 600 nm or 480 nm (FWHM: 10 nm) bandpass filters. The temperature of the solution was controlled during irradiation at 25 ± 0.1°C using an IWAKI CTS-134A constant-temperature system. The gaseous products of photocatalysis, i.e., CO and H_2_, were analyzed by GC-TCD (GL science GC323). A capillary electrophoresis system (Agilent 7100) was used to analyze HCOOH. HPLC analyses for BIH and BI^+^ were conducted using a JASCO 880-PU pump, a Develosil ODS-UG-5 column (250 × 4.6 mm), a JASCO 880–51 degasser, and a JASCO UV-2070 detector. The column temperature was maintained at 30°C using a JASCO 860-CO oven. The mobile phase was a 6:4 (v/v) mixture of acetonitrile and a NaOH–KH_2_PO_4_ buffer solution (50 mM, pH 7) with a flow rate of 0.5 mL·min^−1^.

### Electrochemical Spectroscopy

Electrochemical spectroscopy to determine the molar absorptivity of OERS was performed using a JASCO PU-980 pump and an EC Frontier flow-type electrolysis cell VF-2 equipped with a carbon felt working electrode (18 mm diameter), a Ag/AgNO_3_ (10 mM) reference electrode, and a Pt wire counter electrode in an Ar-saturated acetonitrile solution of **Ru(pic)** (0.5 mM) and Et_4_NBF_4_ (0.1 M) as a supporting electrolyte. Applied potential was controlled using an ALS CHI-720Dx electrochemical analyzer and UV-vis absorption spectra were measured using a Photal MCPD-9800 spectrometer (Otsuka Electronics) and a flow-type transmission cell (light pass length: 1.5 mm) (Ishitani et al., 1994).

### Quantum Yields for One-Electron Reduction of Photosensitizers

A 4-mL DMA–TEOA (5:1 v/v) solution of the photosesnsitizer (0.1 mM) and BIH (0.2 M) in a quartz cubic cell (light pass length: 1 cm) was purged with Ar for 20 min, and then irradiated with the 500-W Xe lamp combined with a 480-nm (FWHM = 10 nm) bandpass filter (Asahi Spectra Co.), ND filter, and a 5-cm-long H_2_O solution filter. UV–vis absorption spectral changes during irradiation were measured using a Photal MCPD-9800 spectrometer (Otsuka Electronics). The light intensity was determined as 5.0 × 10^−9^ einstein·s^−1^ using a K_3_Fe(C_2_O_4_)_3_ actinometer.(Hatchard and Parker, 1956) The amount of OERS of **Ru(pic)** was calculated using the molar absorption coefficient of OERS (500–700 nm) obtained by electrochemical spectroscopy.

## Materials

DMA was dried over molecular sieves 4A, distilled under reduced pressure (~10 mmHg) and used in a week. TEOA was distilled under reduced pressure (<1 mmHg) and used in a month. Both solvents were kept under Ar in the dark. All other reagents were of reagent-grade quality and used without further purification.

### Synthesis

**Ru(dmb)** (Sullivan et al., 1978), **Re** (Morimoto et al., 2013), and BIH (Hasegawa et al., 2005; Zhu et al., 2008) were prepared according to the methods reported in the literatures. **Ru(pic)** was synthesized using a method similar to the synthesis of [Ru(bpy)_2_(pic)](PF_6_) (bpy = 2,2′-bipyridine) (Norrby et al., 1997; Couchman et al., 1998), except for using dmb instead of bpy. [Ru(dmb)_2_(pic)](PF_6_) (**Ru(pic)**): ^1^H NMR (acetone-*d*_6_) δ/ppm: 8.81 (d, *J* = 5.6 Hz, 1H), 8.65 (s, 1H), 8.63 (s, 1H) 8.60 (s, 1H), 8.55 (s, 1H), 8.14 (dd, *J* = 5.6, 0.8 Hz, 1H), 8.03 (dd, *J* = 6.4, 2.4 Hz, 1H), 7.94 (d, *J* = 5.6 Hz, 1H), 7.91 (d, *J* = 5.6 Hz, 1H), 7.77 (d, *J* = 5.6 Hz, 1H), 7.70 (dd, *J* = 5.6, 0.8 Hz, 1H), 7.64 (d, *J* = 5.6 Hz, 1H), 7.50 (dd, *J* = 6.4, 2.4 Hz, 1H), 7.43 (dd, *J* = 5.6, 1.2 Hz, 1H), 7.26 (dd, *J* = 5.6, 1.2 Hz, 1H), 7.21 (dd, *J* = 5.6, 1.2 Hz, 1H), 2.67 (s, 3H), 2.58 (s, 3H), 2.55 (s, 3H), 2.49 (s, 3H). ESI-MS (in acetonitrile) m/z: 592 ([M–PF${}_{6}^{-}$]^+^). Anal. calcd for C_30_H_28_F_6_N_5_O_2_PRu·H_2_O: C, 47.75; H, 4.01; N, 9.28. Found: C, 47.72; H, 3.75; N, 9.40.

## Conclusion

Ruthenium(II) picolinate complex, **Ru(pic)**, successfully functioned as a redox photosensitizer with a much wider wavelength range of visible-light absorption (λ_abs_ < 670 nm) compared with a fairly typical **Ru(dmb)** (λ_abs_ < 560 nm). The system using **Ru(pic)** as a photosensitizer and **Re** as a catalyst photocatalyzed the reduction of CO_2_ to CO by red-light irradiation (λ_ex_ > 620 nm). TON_CO_ reached 235 and Φ_CO_ was 8.0%. Even in the irradiation conditions where **Ru(dmb)** also absorbed light, i.e., λ_ex_ > 500 nm, the system using **Ru(pic)** demonstrated faster CO formation (TOF_CO_ = 6.7 min^−1^) and larger TON_CO_ (2347) than that using **Ru(dmb)** (TOF_CO_ = 3.6 min^−1^, TON_CO_ = 2100).

## Author Contributions

KT, DS, YY, and YT performed all experiments. YU and OI designed this project. YT wrote the manuscript.

### Conflict of Interest Statement

The authors declare that the research was conducted in the absence of any commercial or financial relationships that could be construed as a potential conflict of interest.
