# Supplementary material for: Ruthenium Picolinate Complex as a Redox Photosensitizer With Wide-Band Absorption
Source: Front Chem. 2019 May 14;7:327. doi: 10.3389/fchem.2019.00327 (PMC6527782; doi:10.3389/fchem.2019.00327)
Supplement: Supplementary file 1 [file Data_Sheet_1.PDF]

Supplementary Material

Ruthenium picolinate complex as a redox photosensitizer with wide-band absorption

Yusuke Tamaki[1], Kazuma Tokuda[1], Yasuomi Yamazaki[1,2], Daiki Saito[1], Yutaro Ueda[1], and Osamu Ishitani[1]*

[1]Department of Chemistry, Tokyo Institute of Technology, O-okayama 2-12-1-NE-1, Megruo-ku, Tokyo 152-8550, Japan.

[2]Present address: Department of Materials and Life Science, Seikei University, 3-3-1 Kichijojikitamachi, Musashino, Tokyo 180-8633, Japan.

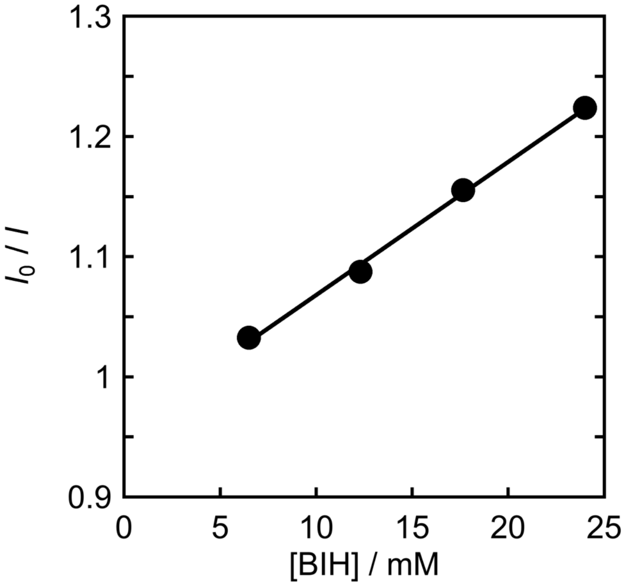

Figure S1. Stern-Volmer plot of emission from **Ru(pic)** quenched by BIH in DMA-TEOA (5:1 v/v). The excitation wavelength was 480 nm.

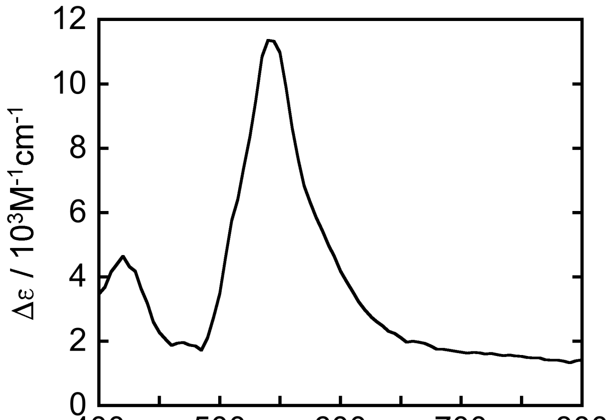

Figure S2. UV-vis absorption spectrum of OERS of **Ru(pic)** in an Ar-saturated acetonitrile solution containing Et_4NBF_4 (0.1 M) obtained by the flow electrolysis with applied potential of -2.05 V vs. $Ag/AgNO_3$.
